# Involvement, depressive symptoms, and their associations with problems and unmet needs in caregivers of adult eating disorder patients

**DOI:** 10.1007/s40519-023-01572-1

**Published:** 2023-05-24

**Authors:** Krisztina Kocsis-Bogar, Michael Ossege, Martin Aigner, Johannes Wancata, Fabian Friedrich

**Affiliations:** 1 Clinical Division of Social Psychiatry, Department of Psychiatry and Psychotherapy, Medical University of Vienna, Vienna Währinger Gürtel 18-20, 1090 Vienna, Austria; 2 Department of Psychiatry and Psychotherapy, Karl Landsteiner University, Krems, Austria

**Keywords:** Eating disorder, Bulimia, Anorexia, Depression, Caregiver burden, Involvement

## Abstract

**Purpose:**

This study aimed to examine the most important problems and needs caregivers of adult inpatients with eating disorders (EDs) are confronted with in their everyday lives. A further aim was to investigate the associations between problems, needs, involvement, and depression in carers.

**Methods:**

Fifty-five caregivers of inpatients with EDs (26 anorexia nervosa, 29 bulimia nervosa) completed the Carers' Needs Assessment, Beck Depression Inventory, and the Involvement Evaluation Questionnaire. The relationships between variables were tested via multiple linear regressions and mediation analyses.

**Results:**

The most frequent problem reported by caregivers was a lack of information about the course and treatment of the illness and consequent disappointment, whereas their most frequently reported needs were different forms of information and counselling. Problems, unmet needs, and worrying were especially high in parents compared to other caregivers. Involvement mediated significantly between problems (*b* = 0.26, *BCa *CI [0.03, 0.49]) as well as unmet needs (*b* = 0.32, *BCa *CI [0.03, 0.59]) of caregivers and their depressive symptoms.

**Conclusion:**

Our findings underline the importance of including the problems and needs of caregivers of adult eating disorder patients in the planning of family and community interventions, to support their mental health.

**Level of evidence:**

Level III: Evidence obtained from cohort or case–control analytic studies.

**Supplementary Information:**

The online version contains supplementary material available at 10.1007/s40519-023-01572-1.

## Introduction

The challenges and subjective burden of caregivers of eating disorder (ED) patients are comparable to those of patients with other severe mental conditions such as schizophrenia [1, 2] and substance-related disorders [3]. Being the primary caregiver and caring for someone with anorexia nervosa (AN) are considered among the main factors contributing to caregivers' subjective burden [4]. Unmet needs for professional help can additionally contribute to caregivers' psychological distress [5] and negatively affect their mental-health-related quality of life [6]. Caregivers of ED patients reported in the semi-structured Carers' Needs Assessment Interview having difficulty communicating with patients and professionals, being disappointed about the chronicity of the ED and the relapses despite treatment, and worrying about the future of the patient. Previous research has identified unmet needs for information [7] and support, mainly through personal contact with health practitioners [8], in caregivers of adult ED patients. Unmet needs [8] or the number of needed and received interventions have been connected to distress in caregivers, independently of the severity of the ED [9].

The prevalence of an elevated level of depressive symptoms in caregivers of ED patients [10–13] is well known. Parents of ED patients are more likely to have a clinically relevant level of depressive symptoms than parents of non-clinical controls [10]. This can be a disadvantage for the treatment of the ED as Forsberg and colleagues [14] show. They found that carers with fewer depressive symptoms can contribute significantly better to the effective treatment of adolescents than those with more depressive symptoms. The majority of studies assessing caregivers' problems and needs concentrate on caregivers of teenage AN patients and there is a knowledge gap about the burden and depressive symptoms of caregivers for other age and diagnostic groups, such as adults with bulimia nervosa (BN) [15].

One potential risk factor for depression in caregivers is involvement [16, 17] which is especially high in caregivers of ED patients compared to other caregivers such as those of patients with schizophrenia [2]. There is a significant association between involvement and depression in caregivers with EDs [10, 18]. Involvement is a multidimensional construct including very different behaviors and emotional expressions reflecting the contradictory nature of the caregivers' role. The active support and helpful interventions of the caregivers in guarding as well as motivating the patient are included in the supervision and urging subdimensions. A strained interpersonal atmosphere and painful cognitions and concerns about the patient's health and future are measured by the tension and worrying subscales of the Involvement Evaluation Questionnaire [19]. Although there are only a few studies analyzing the connections of these subscales with other dimensions of caregivers' difficulties, tension and worrying are seen as descriptive of caregivers' burdens [2]. Out of the four subscales, worrying had the most significant association with mental-health-related quality of life, whereas all four subscales were approximately equally connected to physical-health-related quality of life in the study of Martin (2011). Although tension and worrying seem to be logically similar constructs to depressive symptoms, there is no evidence of the exclusive connection of these two subscales with depressive symptoms in caregivers of ED patients. Contrary to that, Padierna [18] found a connection between depressive symptoms and all three subdimensions of involvement except worrying. Previous results show differences in the associations of the different involvement subscales and unmet needs in caregivers of schizophrenia patients. In the study of Friedrich et al. [16], unmet needs were only connected to worrying and tension.

Previous studies connect over-involvement to unmet needs [16, 17] as well as depressive symptoms [17] in caregivers of schizophrenia patients. The association of needs with psychological distress [9] or burden [5] is known in caregivers of ED patients. Further, a connection between caregivers' burden and maladaptive coping strategies [5] or in other terms between negative experience of caregiving and mental health problems [12] has been shown. It is therefore logical to assume that problems in caring for the patient as well as caregivers' unmet needs are associated with maladaptive coping strategies such as involvement, which is in turn associated with depressive symptoms of caregivers of ED adult patients. According to our knowledge, there is no evidence of this mediation. Additionally, previous studies have mainly investigated the connection between depressive symptoms and involvement as a global construct, but no additional attention has been paid so far to the connection between depressive symptoms and the specific subdimensions of involvement in caregivers of adult ED patients.

Apart from problems and unmet needs, several additional factors have been associated with caregivers' burden in ED, such as time spent with the patient [20], shorter [8] or longer duration of illness [11], the sex of the caregiver, and the family relationship between caregiver and patient. Mothers tend to report more problems [7], more involvement, and a more negative perception of caregiving than fathers or male caregivers [2, 10, 12]. Mothers and female caregivers show higher levels of depressive symptoms [11, 21, 22] than fathers and male caregivers. There are contradictory results regarding the impact of the age of caregivers on their burden. There is evidence of older caregivers showing a higher burden [21] that may be explained by their longer history of caring [11], but some studies found an association between a shorter duration of illness and a more negative experience of caregiving [7].

The present study had multiple aims. The first goal was to investigate if there are differences in the problems and needs, the depressive symptoms, and the involvement of caregivers of AN and BN patients. Based on previous results [4], we expected that caregivers of AN patients would report more burden than caregivers of BN patients. A further aim was to examine if involvement as a global construct as well as the specific dimensions of involvement are significantly connected to depressive symptoms in caregivers of adult AN and BN patients while adjusting for sex, age, and the nature of the kinship of caregivers to patients, as well as the duration of illness. We expected depressive symptoms and involvement to be connected, regardless of the above-mentioned additional variables. Regarding the multidimensional nature of involvement, we expected mainly the dimensions of tension and worrying to be positively connected with depressive symptoms. Further, we hypothesized caregivers' problems and unmet needs to be related to involvement as well as to the specific dimensions of tension and worrying, while adjusting for depressive symptoms, sociodemographic and kinship factors, as well as the duration of illness. A final objective was to investigate the potential mediating role of caregivers' involvement between depressive symptoms and problems as well as unmet needs, respectively.

EDs often require inpatient treatment, which does not only involve high treatment costs, but it has both advantages and disadvantages for the informal caregivers of the patients. The admission into the hospital may be a relief for the carers, who in turn may feel excluded from the treatment and unprepared for the patients' returning home [23]. The objective of this study was therefore to concentrate on in-patients and their caregivers in order to gain some information to prepare low-threshold interventions to support patients and caregivers in preventing further hospitalizations and being able to make more use of outpatient settings.

## Method

### Participants

This study included caregivers of patients with a diagnosis of AN or BN (ICD-10: F50.0-F50.3). Patients were recruited from a ward of the Clinical Division of Social Psychiatry at the Medical University of Vienna specializing in eating disorders and somatoform disorders. Patients’ diagnoses were made by psychiatrists with sufficient clinical experience regarding eating disorders. Patients were asked to name one caregiver with the most significant role in supporting and caring for the patient. Inclusion criteria for the caregivers were a minimum age of 18 years and living in the same household or having face-to-face contact with the patient at least once a week. Patients and caregivers were invited to participate in the study in the order in which patients were admitted to the ward. Psychiatrists, psychiatrists in training, and psychologists performed all interviews and the collection of data. Overall, 65 patients with AN (29) and BN (30) were invited to the study. Unfortunately, only 55 caregivers (26 of AN and 29 of BN patients) agreed to participate, which means an attrition rate of 15%.

### Measures

The *Carers' Needs Assessment* (*CNA-S*) [24] is a semi-structured interview originally developed to assess the problems and needs of caregivers of schizophrenia patients. It has been proven to have sufficiently good content validity, concurrent validity, test–retest reliability, and interrater reliability [24]. The interview assesses 18 problem areas and 15 needed intervention groups in the last 3 months (for a more detailed description see Wancata et al. [24]). It is intended for the use of experts in the treatment of patients with ED (see Graap et al. [7]) as well as by the caregivers themselves. The advantage of this assessment is that it differentiates between problems experienced by the caregiver and the interventions they need to alleviate or limit these problems. Further, the CNA-S assesses the subjective view of caregivers separately from that of professionals. Previous research [1, 7] has shown this interview to be adequate for measuring the problems and needs of caregivers of patients with eating disorders and having a high concordance between the experts’ ratings and caregivers' self-ratings. A similarly high concordance (problems κ ≥ 0.93, *p* < 0.001, *p,* unmet needs κ ≥ 0.79, *p* < 0.001) was found in the present sample, therefore only the self-ratings were included in the analyses. The CNA-S problem areas (α = 0.98), as well as the CNA-S unmet needs (α = 0.83), had good internal consistency in the present sample.

The *Involvement Evaluation Questionnaire* (*IEQ)* [19] is a 31-item self-report questionnaire assessing the care the caregiver gives the patient, personal problems between the caregiver and the patient, as well as the caregiver's worries, coping ability, and subjective burden. Twenty-seven items (scored on a five-point Likert scale of 0 to 4) can be summarized in four subscales. The overall internal consistency of the IEQ was good (α = 0.85) but only the subscales *tension* (α = 0.80) and *worrying* (α = 0.82) were found to be reliable. The internal consistency for *urging* (α = 0.59) and *supervision* (α = 0.45) was poor, for which reason these were not used in the analyses.

The *Beck Depression Inventory* (*BDI)* [25] is a well-established self-report questionnaire containing 21 items that measure depressive symptoms on a four-point Likert scale (from 0 to 3). It is a screening as opposed to a diagnostic tool; however, a score of 18 and above is regarded as clinically relevant for depression. The BDI had good internal consistency in the present sample (α = 0.84).

### Statistical analyses

Data were analyzed using IBM SPSS 26. Patients with AN and BN and their caregivers were compared along continuous variables using the *t*-test. Continuous variables were compared using Welch's test in case of a lack of homogeneity. χ^2^ test was applied to compare categorical variables. In case of expected frequencies lower than five, Fisher's exact test was administered. Multiple linear regression analyses were used to test potential connections between depressive symptoms, involvement, and the problems and unmet needs of caregivers, adjusting for sex, age, and the relationship of caregivers to the patient, as well as the duration of illness. The first regression analysis was run to test associations of depressive symptoms with tension and worrying, the only reliable subscales of IEQ. In regression analyses 2–4 the potential connections of involvement, tension, and worrying with problems and unmet needs were examined, adjusting for depressive symptoms and the above-mentioned additional variables. Finally, the potential mediating role of involvement between caregivers' problems and depressive symptoms, as well as caregivers' unmet needs and depressive symptoms was examined using SPSS PROCESS macro. Missing values were not substituted, because these were in the final sample negligible for most variables (under 2%) or missing systematically when a participant failed to fill in the whole scale. The level of significance was set at *p* ≤ 0.05. The Bonferroni correction was used to reduce the likelihood of Type I errors due to multiple testing.

## Results

The majority of the patients (83.6%) were female and the mean age was 29.47 (*SD* = 9.11). The mean age of caregivers was 43.87 (*SD* = 13.61), and the majority of caregivers (63.6%) were female. According to the group comparisons, AN and BN patients or their caregivers showed no significant differences in any of the examined variables (Table 1).

**Table 1:** Descriptive variables of the whole sample (*N* = 55) and comparison of AN (*n* = 26) and BN (*n* = 29) patients

	Total	AN	BN	Statistics	***p***
	Patients				
	*N* = 55	*n* = 26	*n* = 29		
	Mean (*SD*)	Mean* (SD)*	Mean (*SD*)		
Age	29.47 (9.11)	27.62 (8.35)	31.14 (9.58)	*t* = − 1.44	0.26
Sex	Female: 46 (83.6%)	Female: 22 (84.6%)	Female: 24 82.8%	χ^2^ = 0.04	1.00^a^
Duration of illness	10.11 (8.10)	8.80 (6.64)	11.30 (9.17)	*t* = − 1.15	0.26

	Caregivers				
	Mean (*SD*)	Mean (*SD*)	Mean (*SD*)		
Age	43.87 (13.61)	46.08 (12.64)	41.90 (14.35)	*t* = 1.14	0.26
Sex	Female: 35 (63.6%)	Female: 19 (73.1%)	Female: 16 (55.2%)	Χ^2^ = 1.90	0.14
Relationship to patient				Χ^2^ = 3.43	0.06
Parents	27 (52.9%)	16 (66.7%)	16 (59.3%)		
Other caregiver	24 (47.1%)	8 (33.3%)	11 (40.7%)		
Beck Depression Inventory	8.08 (6.06)	9.33 (5.79)	6.92 (6.18)	*t* = 1.42	0.16
Involvement Evaluation Questionnaire	23.18 (12.62)	24.56 (10.94)	22.04 (13.93)	*t* = 0.71	0.48
Involvement Evaluation Questionnaire tension	6.74 (5.30)	6.60 (4.07)	6.86 (6.24)	*t* = − 0.18	0.86
Involvement Evaluation Questionnaire worrying	11.15 (5.81)	12.33 (5.16)	10.17 (6.21)	*t* = 1.36	0.18
Carers' Needs Assessment Problems	10.89 (4.93)	10.04 (4.64)	9.21 (3.83)	*t* = 0.73	0.47
Carers' Needs Assessment Unmet needs	6.00 (3.78)	6.15 (3.95)	5.86 (3.69)	*t* = 0.28	0.78

^a^Fisher's exact

Because of the inclusion of a relatively heterogeneous caregiver group, the eight different caregiver categories (mother, father, sister, brother, partner, daughter, and others) were dichotomized into parents or other caregivers (i.e. not parents) and compared along the examined variables. Parents were significantly older (*t*(48) = − 6.96 *p* < 0.001) and reported significantly more problems *(t* (48) = − 3.04, *p* = 0.004) than other caregivers. These two differences were the only ones that remained significant after correcting for multiple testing. Parents also reported higher levels of worrying (Welch's *F* (1, 36.88) = 8.94, *p* = 0.005), more unmet needs (*t*(48) = − 2.42, *p* = 0.02), and were more often involved in the care of AN than BN patients (χ^2^ = 4.15, *p* = 0.04) than other caregivers, but these differences lost their significance after the Bonferroni correction (Table 2). Further results of the different specific groups of caregivers are presented in a table of descriptive values in Online Resource 1.

**Table 2:** Comparison of parents and other caregivers along the main variables

	Age Mean (*SD*)	Problems Mean (*SD*)	Unmet needs Mean (*SD*)	Depressive symptoms Mean (*SD*)	IEQ total Mean (*SD*)	IEQ^a^ Tension Mean (*SD*)	IEQ^a^ Worry Mean (*SD*)	DG of patient
Parents total	52.93 (8.46) (*n* = 27)	11.59 (3.69) (*n* = 27)	7.52 (3.13) (*n* = 27)	8.56 (5.43) (*n* = 27)	25.58 (10.64) (*n* = 26)	6.78 (4.76) (*n* = 27)	13.46 (4.19) (*n* = 26)	AN = 16 BN = 11
Other caregivers total	33.78 (10.98) (*n* = 23)	8.39 (3.74) (*n* = 23)	5.09 (3.98) (*n* = 23)	7.37 (6.86) (*n* = 19)	20.50 (15.06) (*n* = 22)	6.52 (6.26) (*n* = 23)	8.74 (6.47) (*n* = 23)	AN = 7 BN = 16
Difference parents-other caregivers	***t*(48) = ** **− 6.96**	***t*(48) = − 3.04**	*t*(48) = − 2.42	*t*(44) = − 0.66	Welch's *F* (1,36.94) = 1.76	*t*(48) = − 0.16	Welch's *F* (1,36.94) = 8.94	Χ = 4.15
***p***	** < 0.001**	**0.004**	0.02	0.52	0.19	0.87	0.005	0.04

^a^Involvement Evaluation Questionnaire. Bold: significant after Bonferroni correction: *p* < .005

The most frequently reported problems and unmet needs of caregivers are listed in Table 3. The most common problem caregivers reported was feeling disappointed about the chronic nature of the illness and the relapses despite treatment. Further, they complained of a lack of relevant information and the additional burden of minor children or siblings of the patients. These problems affected over 70% of the caregivers. The most frequent unmet need was more information on the ED, specifically in the form of psychoeducation, printed material, or counselling (Table 3).

**Table 3:** Problems and unmet needs in the whole sample (*N* = 55)

Problems	Frequency problems (%)	*N*	Intervention categories of unmet needs	Frequency unmet needs (%)	*N*
Disappointment caused by the chronic course of the illness, concerns about the patient’s future	87.3	48	Individual Psychoeducation	67.3	37
Burden of minor children or siblings of the patient	76.4	42	Group Psychoeducation	54.5	30
Not enough information on relapses and their prevention	70.9	39	Printed information material	49.1	27
Not enough Information on the illness, its symptoms, and its course	69.1	38	Counselling for caregivers	58.2	32
Not enough information on treatment	65.5	36	Family counselling	54.5	30
Not enough information on rehabilitation	63.6	35	Professionally led caregiver group	50.9	28
Communication problems and conflicts with the patient	58.2	32	Case management for patient	49.1	27
Carer's stress due to earlier life events	58.2	32	Psychotherapy, family therapy	34.5	19
Burnout of the carer	58.2	32	Establishing a daily program for patient	32.7	18
Communication problems with the professionals	58.2	32	Self-help group for caregivers	30.9	17
Burden caused by non-compliance or dangerous behavior of the patient	56.4	31	Counselling by social worker	27.3	15
Problems caused by relapses or crises	52.7	29	Temporary supervision of the patient at home by a professional	27.13	15
Financial burden	40	22	Separate accommodation for patient	16.4	9
Social isolation, conflicts within the family	36.4	20	Financial support	14.5	8
Over-involvement, difficulties because patient lives in the same apartment	36.4	20	Diagnosis and/or treatment for carer at a general practitioner	12.7	7
Feelings of guilt, being blamed	32.7	18			
Not enough time for oneself	27.3	15			
Fear of stigmatization and discrimination	12.7	7			

In the linear regression analyses, potential predictors of depressive symptoms (BDI) and involvement as well as the tension and worrying subscales of involvement were examined. Model 1, which examined the predictors of BDI, did not reach significance. Model 2, which analyzed the potential predictors of involvement, was significant. BDI was the only significant predictor, explaining 40% of the variance of involvement, whereas the age, sex, relationship of the caregiver to the patient (parents vs. other caregivers), the duration of illness, and caregivers' problems or unmet needs were not significant. Model 3, which examined the potential connection of IEQ tension to caregivers' depressive symptoms, problems, and unmet needs, adjusted for the length of illness, the age, sex, and relationship of the caregiver to the patient, was not significant. Model 4, which examined the connection of the above predictors to IEQ worrying, reached significance. Depressive symptoms explained 44% of the variance, whereas all the further examined variables remained insignificant (Table 4).

**Table 4:** Predictors of depressive symptoms and involvement, as well as the tension and worrying dimensions of the Involvement Evaluation Questionnaire in the whole sample (*N* = 55)

Dependent variables, model fitness	Independent variables	β	t	Significance	Tolerance	VIF
Model 1 BDI total *R*^*2*^ = 0.23 *F* = 1.97 *p* = 0.09	IEQ^a^ tension	0.10	0.47	0.64	0.35	2.88
	IEQ worrying	0.38	1.84	0.07	0.47	2.12
	Caregivers' sex	0.02	0.16	0.87	0.89	1.13
	Caregivers' age	− 0.19	− 0.77	0.45	0.31	3.25
	Parents/other caregivers	0.05	0.19	0.85	0.30	3.39
	Duration of illness	− 0.11	− 0.59	0.56	0.56	1.80
Model 2 IEQ total *R*^*2*^ = 0.40 *F* = 3.38 *p* = 0.007	CNA^b^-Problems	0.04	0.20	0.84	0.39	2.55
	CNA-Unmet needs	0.29	1.34	0.19	0.37	2.70
	BDI^c^ total	**0.33**	**2.36**	**0.02**	**0.88**	**1.14**
	Caregivers' sex	− 0.03	− 0.22	0.83	0.88	1.14
	Caregivers' age	0.03	0.12	0.91	0.27	3.77
	Parents/other caregivers	0.10	0.39	0.70	0.26	3.88
	Duration of illness	− 0.10	− 0.54	0.59	0.52	1.91
Model 3 IEQ Tension *R*^*2*^ = 0.22 *F* = 1.59 *p* = 0.17	CNA-Problems	− 0.04	− 0.18	0.86	0.32	3.06
	CNA-Unmet needs	0.29	1.20	0.24	0.35	2.84
	**BDI total**	0.25	1.61	0.12	0.86	1.17
	Caregivers' sex	− 0.03	− 0.17	0.87	0.90	1.11
	Caregivers' age	0.16	0.64	0.53	0.31	3.24
	Parents/other caregivers	− 0.26	− 0.98	0.33	0.29	3.48
	Duration of illness	− 0.25	− 1.35	0.19	0.58	1.73
Model 4 IEQ Worrying *R*^*2*^ = 0.44 *F* = 4.32 *p* = 0.001	CNA-Problems	− 0.17	− 0.80	0.43	0.34	2.96
	CNA-Unmet needs	0.40	1.99	0.05	0.38	2.75
	**BDI total**	**0.30**	**2.33**	**0.03**	**0.87**	**1.14**
	Caregivers' sex	− 0.05	− 0.40	0.28	0.88	1.13
	Caregivers' age	0.24	1.11	0.28	0.31	3.24
	Parents/other caregivers	0.70	0.31	0.76	0.29	3.47
	Duration of illness	− 0.27	− 1.68	0.10	0.58	1.71

Significant predictors are bold; ^a^Involvement Evaluation Questionnaire; ^b^Carers' Needs Assessment; ^c^Beck Depression Inventory. Bold: significant after Bonferroni correction: *p* < 0.013

**Fig. 1:**
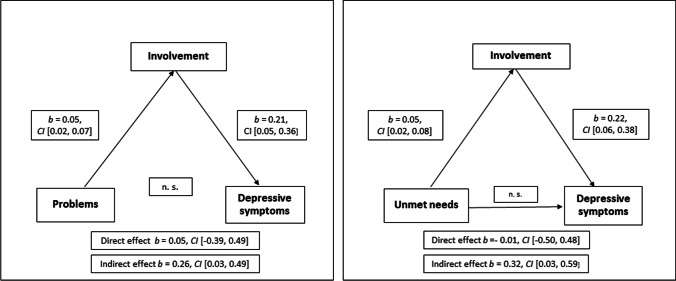
Involvement as a mediator in the relationship between depressive symptoms and problems as well as unmet needs

According to the mediation analyses, caregivers' problems predicted involvement significantly but there was no direct association between problems and depressive symptoms. Involvement had a significant mediating effect between problems and depressive symptoms. Similarly, unmet needs predicted involvement significantly but had no direct association with depressive symptoms. Involvement was a significant mediator between unmet needs and depressive symptoms (Fig. 1).

## Discussion

According to our knowledge, this is the first study to give such an in-depth analysis of the associations between problems, unmet needs, involvement, and depressive symptoms in caregivers of adult ED patients. Caregivers in the present study reported very similar problems as in a previous ones [1, 7], concerning disappointment about the chronic nature of the illness, relapses, and a lack of information about the progress of the disease and treatment. Similarly to the needs reported before [8, 9], they claimed to need more information and several different forms of professional help. The similarity of problems caregivers used to face and are confronted with now probably shows that the challenges of EDs remain the same over time, despite greater societal awareness and improvements in the health care system. It may sound like a promising result that only about a third of the caregivers reported the unmet need for such interventions as psychotherapy or family therapy, a daily program for the patient, or a self-help group for caregivers. It is still alarming that two-thirds of the caregivers require such low-threshold interventions as psychoeducation and half of them need printed information material. Based on this, it can be concluded that certain needs of the majority of caregivers could be met through simple and low-cost measures such as regularly offering flyers and psychoeducation groups in both inpatient and outpatient care.

The major finding of the present study is that contrary to our expectations and evidence in caregivers of schizophrenia patients [17], neither caregivers' problems nor their unmet needs were connected directly to their depressive symptoms. Involvement, however, was significantly connected to depressive symptoms and had a mediating role between depressive symptoms and problems as well as unmet needs. The rationale for this mediation may be similar to that of the connections between unmet needs, involvement, and depressive symptoms in caregivers of schizophrenia patients described by Krautgartner [17]: caregivers with more problems and unmet needs try to compensate for the shortcomings of the healthcare system, get over-involved and overburdened which is a risk factor for developing depressive symptoms.

Parents reported more problems than other caregivers. However, being a parent did not predict either depressive symptoms or involvement in any way. The implication for clinical practice is that the support of caregivers of adult eating disorder patients deserves attention regardless of the nature of the relationship between patient and caregiver. Evidence suggests that the treatment of adult ED patients can be more effective when informal caregivers are involved [26]. Programs transferring information, offering psychoeducation and skills training for caregivers of ED patients like Experienced Carers Helping Others are showing promising results in reducing the burden of caregivers as well as helping ED patients more effectively in the case of teenagers [15, 27]. The effectiveness of such family-based methods in the treatment of adult ED patients should be examined by future studies [15].

Even though less than half of the caregivers of adult eating disorders patients reported living in the same dwelling as the patient, depressive symptoms and involvement were still significantly associated, regardless of the sex or age of the caregivers or their kinship to the patients. Out of the four dimensions of involvement, only worrying could be significantly connected to the depressive symptoms of caregivers. It is important to note that this dimension was specifically found to be strongly prevalent in the caregivers of multiple psychiatric disorders, and it was the highest in caregivers of ED patients compared to those of patients with depression and schizophrenia in the study of Martín and colleagues [2]. Of course, the cross-sectional nature of the present study does not allow us to draw any causal conclusions.

Present findings are contrary to previous ones [7, 11] since neither the duration of illness nor the age of caregivers was found to be significantly connected to depressive symptoms in caregivers. One reason for this difference may be that the majority of these studies investigated only or mainly adolescents or young adults with AN, mostly living together with the patients. The present sample consist of patients of a more mature age and more independence from the caregivers which may reduce the immediate negative effect of the caregiving burden on caregivers' mental health. Unlike several previous studies [22], the present findings do not support a higher level of depressive symptoms or involvement in female than male caregivers or specifically in mothers. This latter result is especially interesting as regards the higher prevalence of depressive symptoms in females. One of the reasons for these negative results may be the relatively large number of predictors in the analyses in relation to the sample size. A further reason may be that clinically relevant BDI scores were only achieved by 14% of the caregivers sampled. Although this proportion is similar to several other studies in the field, e.g. [28], and definitely higher than the point prevalence of depressive disorders measured in the European Union [29] in adults (6.38%) or females only (7.74%), it may still be too small to show an effect in the present small sample. Additionally, it is important to note that by using the BDI depressive symptoms were measured but the BDI does not give a diagnosis of depression according to the ICD or DSM.

The small number of participants must be mentioned as one of the main limitations of the present study, which prevented the inclusion of several important sociodemographic factors such as the marital status of caregivers or the level of functioning of patients in the analyses. It was a mono-center study and the data were collected at a university hospital department from caregivers of inpatients only, which is likely to have reduced the generalizability of the results to caregivers of outpatients and patients with a shorter duration of illness. The fact of getting treatment at a university hospital and the willingness to participate in a study may show an extraordinary motivation and involvement on behalf of the participating caregivers, which may not be applicable to caregivers of patients in other inpatient settings. By reducing the data gained from an interview on the number of problems and unmet needs, a lot of information had to be omitted from the analyses. At the same time, attrition in caregivers was relatively high and the data about caregivers who dropped out are too inconsistent to conduct a reliable analysis and draw valid conclusions. The clinical implications of the study are however that caregivers of adult ED patients tend to have the need to be informed and included in the ED treatment. Fulfilling these needs may reduce their involvement, especially their worrying, in the everyday life of the patients, and may contribute to their better mental health, as well as support their ability to provide the necessary care.

## Strength and limits

The generalizability of the results of the present study is limited by the small number of participants and by the fact that it is a mono-center study carried out in a university hospital. The present findings are however crucial because they highlight the specific needs of caregivers of adult BN as well as AN patients. Further, these results are informative about the mediating role that involvement plays between caregivers' problems or unmet needs and depressive symptoms.

## What is already known on this subject?

The subjective burden in connection with problems and unmet needs in caregivers of ED patients has been well explored. However, most of the previous evidence has been obtained from caregivers of teenage patients with AN and a lot less is known about caregivers of adults, especially those with BN. The connection between problems and unmet needs with psychological distress in caregivers of ED patients has been already shown, but the associations between depressive symptoms and the dimensions of involvement were not clear.

## What does this study add?

According to our results, problems and unmet needs are not directly connected with depressive symptoms but are connected indirectly through involvement. The present findings show a great similarity in caregiver burden in the case of adult AN and BN patients, regardless of whether the caregivers are parents or other family members. These results emphasize the need to provide short interventions to support caregivers of adult AN and BN patients.

## Supplementary Information

Below is the link to the electronic supplementary material.

Supplementary file1 (DOCX 21 KB)

## Data Availability

The data underlying this article will be shared on reasonable request to the corresponding author.
