# Supplementary material for: Involvement, depressive symptoms, and their associations with problems and unmet needs in caregivers of adult eating disorder patients
Source: Eat Weight Disord. 2023 May 24;28(1):45. doi: 10.1007/s40519-023-01572-1 (PMC10209275; doi:10.1007/s40519-023-01572-1)
Supplement: Supplementary file 1 — Supplementary file1 (DOCX 21 KB) [file 40519_2023_1572_MOESM1_ESM.docx]

# Involvement, depressive symptoms and their associations with problems and unmet needs in caregivers of adult eating disorder patients.

**Eating and Weight Disorders: Studies on Anorexia, Bulimia and Obesity**

Kocsis-Bogar, Krisztina^1,*^ **(0000-0003-4188-5047),** Ossege, Michael.^1^, Aigner, Martin^1, 2^, Wancata, Johannes^1^ (0000-0003-4951-6780), Friedrich, Fabian^1^ ([0000-0003-2219-9631](https://orcid.org/0000-0003-2219-9631))

# ^1^: Clinical Division of Social Psychiatry, Department of Psychiatry and Psychotherapy, Medical University of Vienna, Vienna, Austria

# ^2^: Department of Psychiatry and Psychotherapy, Karl Landsteiner University, Krems, Austria

^*:^ Corresponding author: Krisztina Kocsis-Bogar, krisztina.kocsis-bogar@meduniwien.ac.at

Address of correspondence: Clinical Division of Social Psychiatry, Department of Psychiatry and Psychotherapy, Medical University of Vienna, A-1090 Vienna Währinger Gürtel 18-20

**Supplementary Table 1** Descriptives of the different caregivers in the whole sample

|  | Age  Mean  (*SD*) | Problems  Mean  (*SD*) | Unmet needs  Mean  (*SD*) | Depressive symptoms  Mean  (*SD*) | IEQ total  Mean  (*SD*) | IEQ^a^ Tension  Mean  (*SD*) | IEQ^a^ Worry  Mean  (*SD*) | DG of patient |
| --- | --- | --- | --- | --- | --- | --- | --- | --- |
| Mothers | 51.65 (7.69) (*n* = 20) | 11.65 (3.82)  (*n* = 20) | 7.40  (3.22)  (*n* = 20) | 8.75  (5.34)  (*n* = 20) | 25.65 (10.73)  (*n* = 20) | 6.40 (4.36)  (*n* = 20) | 13.30  (4.18)  (*n* = 20) | AN = 13  BN = 7 |
| Fathers | 56.57 (10.10)  (*n* = 7) | 11.42 (3.60)  (*n* = 7) | 7.86 (3.08)  (*n* = 7) | 8  (6.08)  (*n* = 7) | 25.33 (11.33)  (*n* = 6) | 7.86 (6.01)  (*n* = 7) | 14  (4.56)  (*n* = 6) | AN = 3  BN = 4 |
| Sisters | 44.50 (3.54)  (*n* = 2) | 9  (1.41)  (*n* = 2) | 9  (2.83)  (*n* = 2) | 2  (-)  (*n* = 1) | 9  (1.41)  (*n* = 2) | 3  (0)  (*n* = 2) | 5.50  (0.71)  (*n* = 2) | AN = 0  BN = 2 |
| Brothers | 31  (6.25)  (*n* = 3) | 9  (2)  (*n* = 3) | 5.67 (3.06)  (*n* = 3) | 11.67 (9.45)  (*n* = 3) | 17  (17.44)  (*n* = 3) | 8.33 (9.23)  (*n* = 3) | 9.33  (9.45)  (*n* = 3) | AN = 0  BN = 3 |
| Partners | 38.30 (11.22)  (*n* = 10) | 9.10  (4.98)  (*n* = 10) | 5.20 (4.37)  (*n* = 10) | 7.25  (6.50)  (*n* = 8) | 22.56  (11.44)  (*n* = 9) | 6.60 (6.83)  (*n* = 10) | 8.30  (4.90)  (*n* = 10) | AN = 4  BN = 6 |
| Daughters | 19.33 (1.53)  (*n* = 3) | 9.33 (2.08)  (*n* = 3) | 4  (5.20)  (*n* = 3) | 3.67  (4.73)  (*n* = 3) | 19  (18)  (*n* = 3) | 7  (7)  (*n* = 3) | 8.33  (8.50)  (*n* = 3) | AN = 1  BN = 2 |
| Others | 30.80 (8.59)  (*n* = 5) | 5.8  (2.39)  (*n* = 5) | 3.6  (3.51)  (*n* = 5) | 8.5  (8.10)  (*n* = 4) | 24.40 (22.58)  (*n* = 5) | 6.40  (5.86)  (*n* = 5) | 10.80 (9.15)  (*n* = 5) | AN = 2  BN = 3 |

*Note.* ^a^: Involvement Evaluation Questionnaire.
